# Underlying Disorders, Clinical Phenotypes, and Treatment Diversity among Patients with Disseminated Intravascular Coagulation

**DOI:** 10.31662/jmaj.2020-0023

**Published:** 2020-09-23

**Authors:** Hiroyuki Ohbe, Kazuma Yamakawa, Kohei Taniguchi, Kojiro Morita, Hiroki Matsui, Kiyohide Fushimi, Hideo Yasunaga

**Affiliations:** 1 Department of Clinical Epidemiology and Health Economics, School of Public Health, University of Tokyo, Tokyo, Japan; 2 Division of Trauma and Surgical Critical Care, Osaka General Medical Center, Osaka, Japan; 3 Translational Research Program, Osaka Medical College, Osaka, Japan; 4 Department of Health Policy and Informatics, Tokyo Medical and Dental University Graduate School of Medicine, Tokyo, Japan

**Keywords:** bleeding, disseminated intravascular coagulation, Japan, phenotype, observational study, organ failure

## Abstract

**Introduction:**

Clinical guidelines state that disseminated intravascular coagulation (DIC) treatment should be based on three clinical phenotypes: the marked bleeding type (e.g. leukemia, trauma, obstetric diseases, or aortic diseases); organ failure type (sepsis or pancreatitis); and asymptomatic type of DIC (solid cancer). However, among the various underlying disorders of DIC, the clinical presentations of bleeding or organ failure have not to date been well documented. The present study aimed to evaluate whether underlying disorders of DIC would affect clinical outcome including death, organ failure, and bleeding.

**Methods:**

Using the Japanese Diagnosis Procedure Combination inpatient database, we identified all adult patients diagnosed with DIC during hospitalization from July 1, 2010, to March 31, 2018. We collected data on patient characteristics and underlying disorders of DIC including sepsis, solid cancer, leukemia, trauma, obstetric diseases, aortic diseases, pancreatitis, and miscellaneous diseases. We counted major bleeding events and calculated an organ failure score for patients during hospitalization.

**Results:**

We identified 337,132 patients with DIC. The major disorders underlying DIC were sepsis (42%) and solid cancer (31%). The average organ failure scores of patients with aortic diseases, sepsis, and trauma were 2.8, 2.2, and 2.2, respectively. The percentages with major bleeding events among patients with aortic diseases, trauma, obstetric diseases, and solid cancer were 24%, 15%, 10%, and 10%, respectively.

**Conclusions:**

This study suggests that the clinical presentations of bleeding and organ failure are not associated with the three existing clinical phenotypes of DIC or with the underlying disorders of DIC. Therefore, clinical presentation alone may not be sufficient for identifying the clinical phenotypes of DIC. Further research is necessary to develop new strategies for identifying the phenotypes of DIC and improving treatment strategies for individual patients.

## Introduction

Disseminated intravascular coagulation (DIC) is a life-threatening condition which can result in organ failure as well as severe bleeding because of decreased platelets and coagulation factors, which are characteristic of the systemic activation of widespread thrombosis ^(1), (2), (3)^. DIC never occurs in isolation, and there are always underlying disorders including sepsis, malignancy, trauma, obstetric diseases, vascular anomalies, toxins, or immunological abnormalities ^(1), (2), (3), (4), (5)^. While all these disorders can induce the systemic activation of coagulation, the degree of fibrinolysis activation may vary. DIC may be classified into three clinical phenotypes which clinical guidelines state that DIC treatment should be based on ^(2), (6), (7), (8)^. These clinical phenotypes are marked bleeding, organ failure, and asymptomatic ^(6), (9)^. 

Currently, the clinical phenotype of DIC for a particular case is largely determined using information on the disorders underlying DIC ^(6)^. However, the clinical presentations of bleeding and organ failure are not well understood among the various underlying disorders of DIC. A previous small-scale observational study including 204 patients with DIC who had various underlying disorders showed that the incidence rates of bleeding and organ failure varied depending on the underlying disorders, which overlapped in complicated ways, but the study included fewer than 10 patients for most of the underlying disorders examined ^(10)^. Furthermore, no study to date has evaluated treatment patterns of DIC based on the underlying disorders or phenotypes.

The clinical presentations of bleeding and organ failure may not be consistent for the same underlying disorders, and they may also shift or change over time ^(11)^. Moreover, the heterogeneity of the underlying disorders and their clinical presentation makes it difficult to find an appropriate therapeutic approach for an individual patient with DIC ^(1)^. Therefore, using a nationwide inpatient database in Japan, the present study aimed to evaluate whether underlying disorders of DIC would affect clinical outcome including death, organ failure, and bleeding. We also evaluated the treatment diversity based on the underlying disorders of DIC. Based on the heterogeneity of the underlying disorders and their clinical presentation, these clinical features may provide further insight into individualized treatment strategies.

## Materials and Methods

This was a retrospective observational study using routinely collected data. The study was approved by the Institutional Review Board of The University of Tokyo (approval number: 3501-[1] [July 25, 2011]). Because of the anonymous nature of the data, the Board waived the requirement for informed consent: No information on individual patients, hospitals, or treating physicians was obtained.

### Data source

We used the Japanese Diagnosis Procedure Combination inpatient database, which includes discharge abstracts and administrative claims data for more than 1,200 acute-care hospitals and covers approximately 90% of all tertiary-care emergency hospitals in Japan. The database includes data on age, sex, primary diagnoses, concomitant diagnoses, complication diagnoses, procedures, prescriptions, and discharge status. In the database, diagnoses are recorded using International Classification of Diseases, Tenth Revision (ICD-10) codes and text in the Japanese language. For the purposes of treatment cost reimbursement, attending physicians are required to report objective evidence for their diagnoses because the diagnostic records are linked to a payment system. With diagnostic sensitivity and specificity of 35.8% and 98.2%, respectively, previous study established the validity of the diagnosis of DIC in this database ^(12)^. The sensitivity and specificity of treatments and procedures in the database have been reported to exceed 90% ^(13)^.

### Study population

We identified all adult patients diagnosed with DIC during hospitalization from July 1, 2010, to March 31, 2018. All hospitalized patients who were diagnosed with DIC (ICD-10 codes: D65, O450, O460, O723, O081) in the primary, concomitant, or complication diagnoses were included in the study. This means that the DIC diagnoses used in this study were the attending physicians’ recorded diagnoses, which are assumed to be based on the diagnostic criteria from the Japanese Association for Acute Medicine or the International Society of Thrombosis and Hemostasis ^(2), (14)^.

All patients diagnosed with DIC were identified and categorized as having one of nine underlying disorders, which were predefined on the basis of prior reviews and existing guidelines ^(2), (6), (8), (11), (14), (15)^: sepsis solid cancer; leukemia; trauma; obstetric diseases; aortic diseases; pancreatitis; miscellaneous disorders; and undetermined. The classification process used a hierarchical system of diagnoses and procedures to create mutually exclusive groups (see Supplementary Table S1 for additional details). These underlying disorders were further classified into three clinical phenotypes of DIC on the basis of previous studies: 1) marked bleeding type (leukemia ^(16)^, trauma ^(17)^, obstetric diseases ^(18)^, and aortic diseases ^(19)^); 2) organ failure type (sepsis ^(20)^ and pancreatitis ^(21)^); and 3) asymptomatic type (solid cancer ^(22)^).

We excluded patients who had a suspected diagnosis of DIC and patients who were younger than 18 years of age. We excluded the second and all subsequent admission records for patients who were admitted with a diagnosis of DIC more than once during the study period. We also excluded patients with Child-Pugh Class C cirrhosis and those with an undetermined category of disease underlying DIC.

### Study variables

We collected the following baseline patient characteristics: age; sex; Charlson comorbidity index ^(23)^; planned or emergency admission; intensive care unit admission; admission to a high-care unit; surgery with general anesthesia; use of antibiotics; annual hospital volume; underlying disorders of DIC; type of infection; type of solid cancer; use of anticoagulants for DIC; use of antifibrinolytics for DIC; and use of blood components for DIC. Type of infection and type of solid cancer were identified by using ICD-10 codes listed in Supplementary Table S2 and Supplementary Table S3, respectively. Anticoagulants used for DIC treatment included antithrombin, recombinant human soluble thrombomodulin, serine protease inhibitors (gabexate mesylate or nafamostat mesilate), and heparin (≥10,000 units of unfractionated heparin or low-molecular-weight heparin). Antifibrinolytics used for DIC treatment included intravenous tranexamic acid, and blood components used for DIC treatment included fresh frozen plasma and platelets.

We calculated an organ failure score, following a previously published method for identifying the severity of illness using patient organ failure based on ICD-10 codes and procedure codes as an indicator of the organ failure type of DIC ^(24)^. This score encompassed data on cardiovascular, respiratory, neurologic, hematologic, hepatic, and renal organ failure, and the details of the ICD-10 codes and Japanese procedure codes used are shown in Supplementary Table S4.

As an indicator of the marked bleeding type of DIC, we defined major bleeding events with the ICD-10 codes for intracranial bleeding (I60-I62, S064-S066), major gastrointestinal bleeding (I850, I864, K250, K252, K254, K256, K260, K262, K264, K266, K270, K272, K274, K276, K280, K282, K284, K286, K625, K920-K922), respiratory bleeding (J942, R04), renal/urinary tract bleeding (R31), ocular bleeding (H313, H356, H431, H450), retroperitoneal bleeding (K661), pericardial bleeding (I312), and bleeding attributed to anemia (D500, D62). This definition has been used previously ^(25)^.

We also collected data on the following general outcomes: in-hospital mortality; length of hospital stay; and total hospitalization cost.

### Statistical analysis

Categorical variables are presented as numbers and percentages. Continuous variables are presented as means and standard deviations or medians and interquartile ranges. All analyses were performed using Stata/MP, Version 16.0 (StataCorp, College Station, TX, USA).

## Results

**Figure 1.**
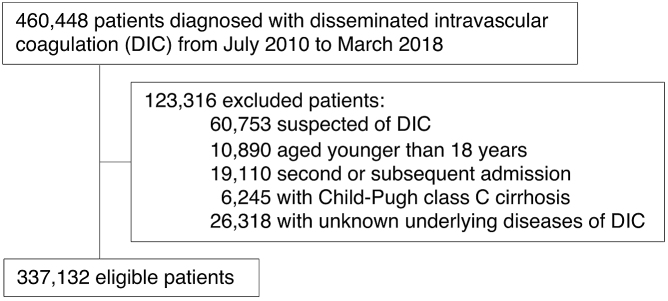
Patient flowchart for inclusion in the study.

After applying the inclusion and exclusion criteria, a total of 337,132 patients were included in the present study (Figure 1). Table 1 shows the patient characteristics. The average age was 71 years, and 55% of the patients were male. Most cases of DIC had the underlying disorders of sepsis (42%) or solid cancer (31%). As anticoagulants for DIC treatments, antithrombin, recombinant thrombomodulin, serine protease inhibitors, and heparin were used in 25%, 36%, 37%, and 30% of the patients, respectively. For antifibrinolytics and blood components, tranexamic acid, fresh frozen plasma, and platelets were used in 14%, 26%, and 25% of the patients, respectively. The overall in-hospital mortality was 37%. The average organ failure score was 2.0, and major bleeding events were observed in 9.3% of the patients.

**Table 1.** Patient Characteristics.

	Total
	(n = 337,132)
Age (years), mean (SD)	71 (17)
Male, n (%)	183,928 (55)
Charlson comorbidity index, mean (SD)	1.4 (1.9)
Planned or emergency admission, n (%)	
Planned admission	172,754 (51)
Emergency admission	164,378 (49)
Intensive care unit admission, n (%)	89,316 (26)
High-care unit admission, n (%)	55,066 (16)
Surgery with general anesthesia, n (%)	88,523 (26)
Use of antibiotics, n (%)	314,033 (93)
Annual hospital volume (patients per year), mean (SD)	76 (52)
Underlying disorders, n (%)	
Marked bleeding type of DIC	
Leukemia	31,059 (9)
Trauma	16,844 (5)
Obstetric diseases	16,742 (5)
Aortic diseases	9,721 (3)
Organ failure type of DIC	
Sepsis	140,538 (42)
Lung	37,847 (11)
Abdomen	41,224 (12)
Urinary tract	26,733 (8)
Central nervous system	3,274 (1)
Skin and soft tissues	4,882 (2)
Cardiovascular system	6,429 (2)
Other infections	38,090 (11)
Pancreatitis	9,052 (3)
Asymptomatic type of DIC	
Solid cancer	104,323 (31)
Esophagus	3,749 (1)
Stomach	17,911 (5)
Colon	19,816 (6)
Liver	9,578 (3)
Bile duct/gallbladder	10,188 (3)
Pancreas	10,707 (3)
Lung	10,816 (3)
Gynecological	4,874 (1)
Urological	10,981 (3)
Breast	2,575 (1)
Other solid cancers	12,503 (4)
Miscellaneous	8,853 (3)
Anticoagulants used for DIC, n (%)	
Antithrombin	85,488 (25)
Recombinant thrombomodulin	122,224 (36)
Serine protease inhibitors	125,344 (37)
Heparin	102,048 (30)
Antifibrinolytics used for DIC, n (%)	
Tranexamic acid	47,408 (14)
Blood components used for DIC, n (%)	
Fresh frozen plasma	87,098 (26)
Platelets	82,959 (25)
Organ failure score, mean (SD)	2.0 (1.0)
Major bleeding events, n (%)	33,454 (10)
Outcomes	
In-hospital mortality, n (%)	125,933 (37)
Length of stay (days), mean (SD)	41 (83)
Total hospitalization costs (thousands of USD), mean (SD)	25 (31)

SD: standard deviation; DIC: disseminated intravascular coagulation; USD: United States Dollar

**Figure 2.**
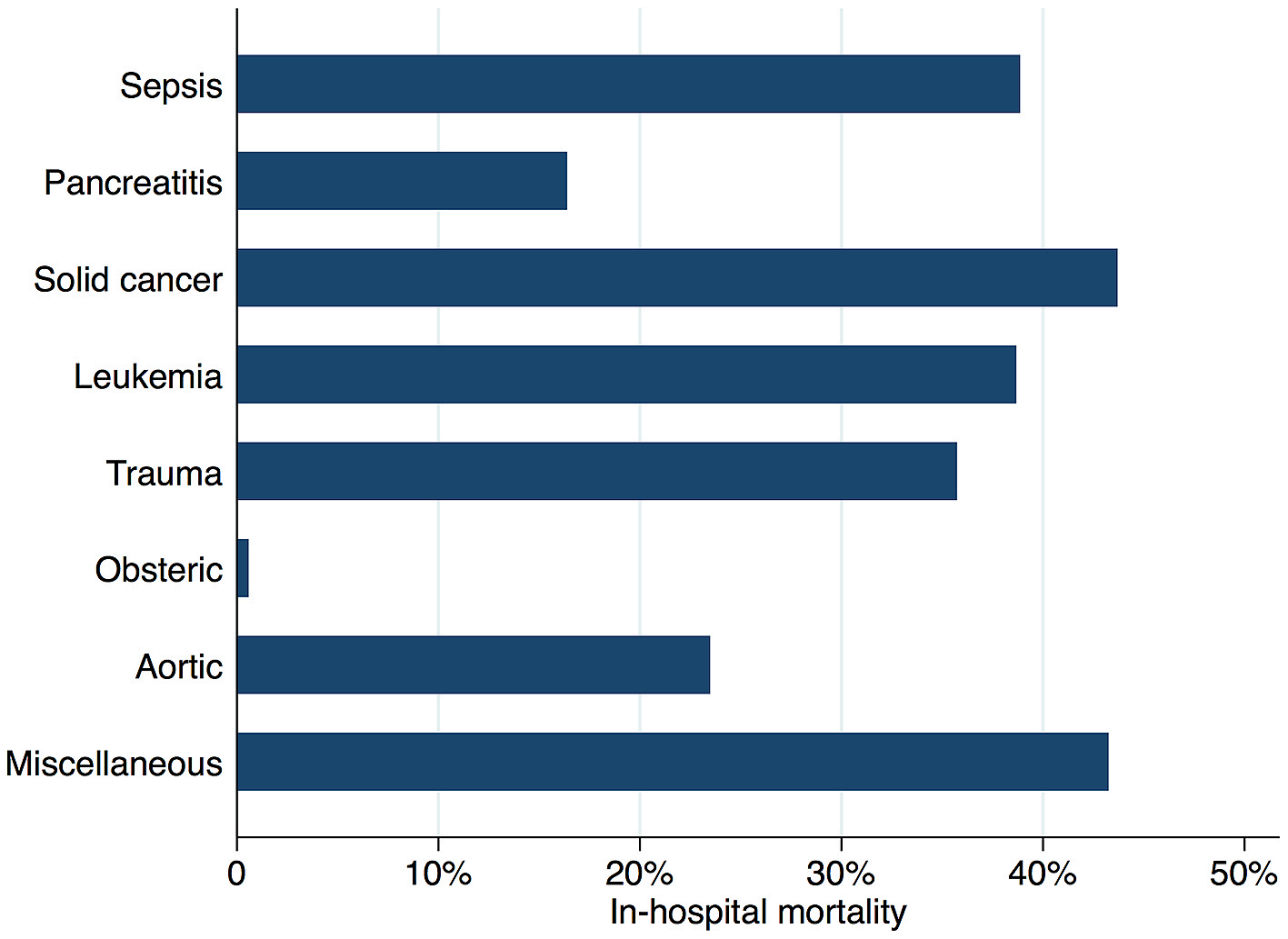
In-hospital mortality by underlying disorders of disseminated intravascular coagulation.

**Figure 3.**
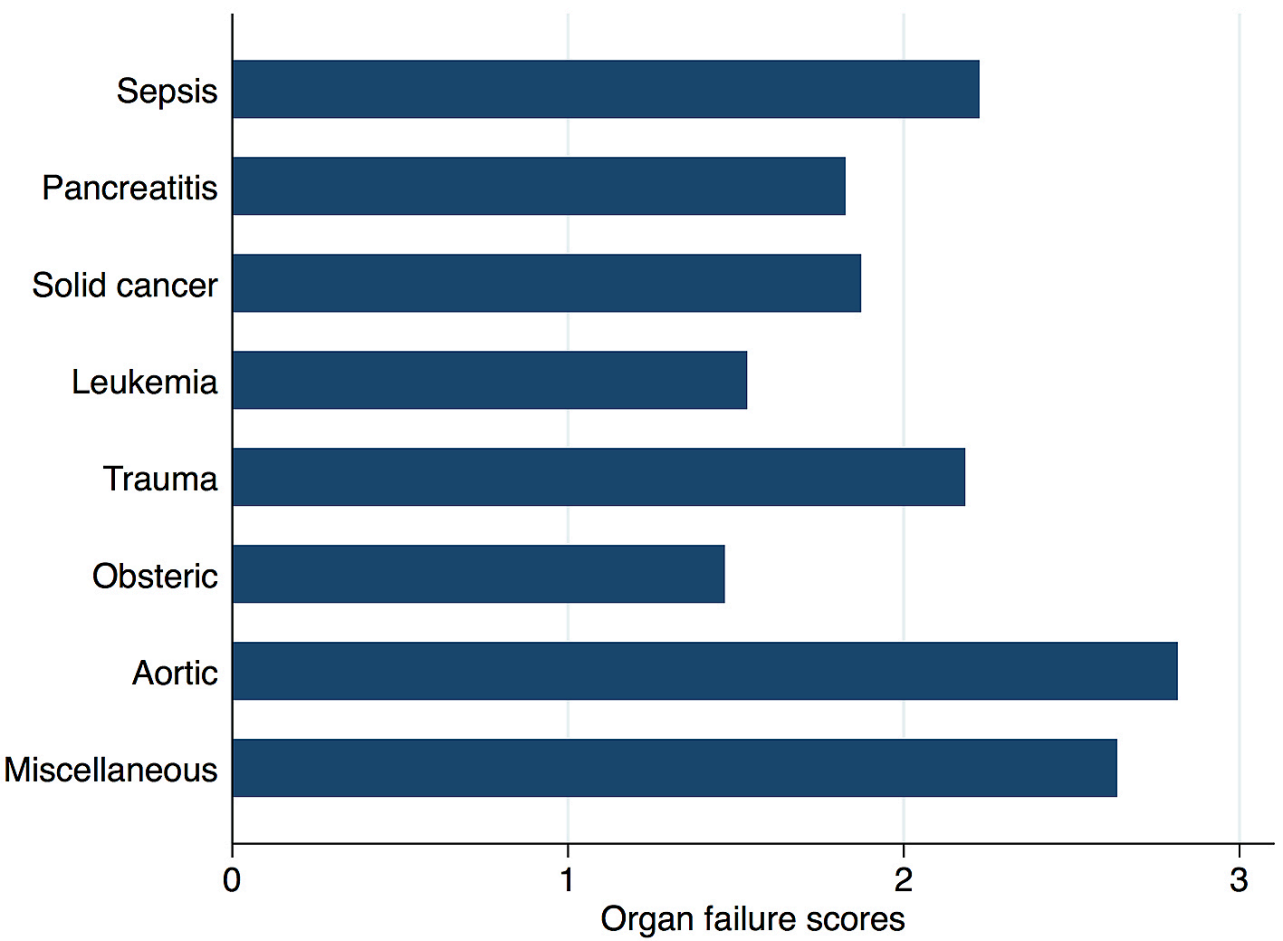
Organ failure score by underlying disorders of disseminated intravascular coagulation.

**Figure 4.**
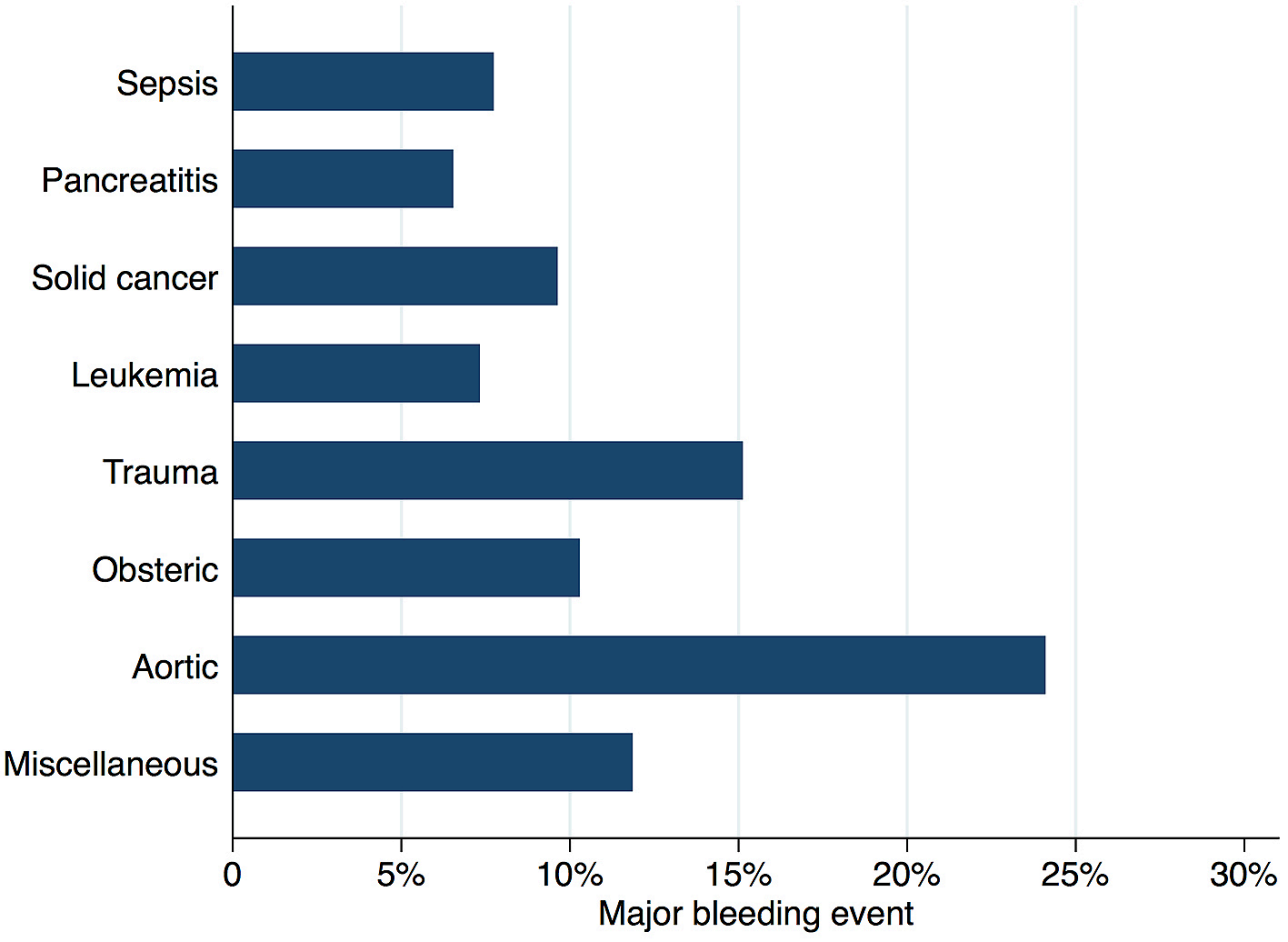
Major bleeding events by underlying disorders of disseminated intravascular coagulation.

**Figure 5.**
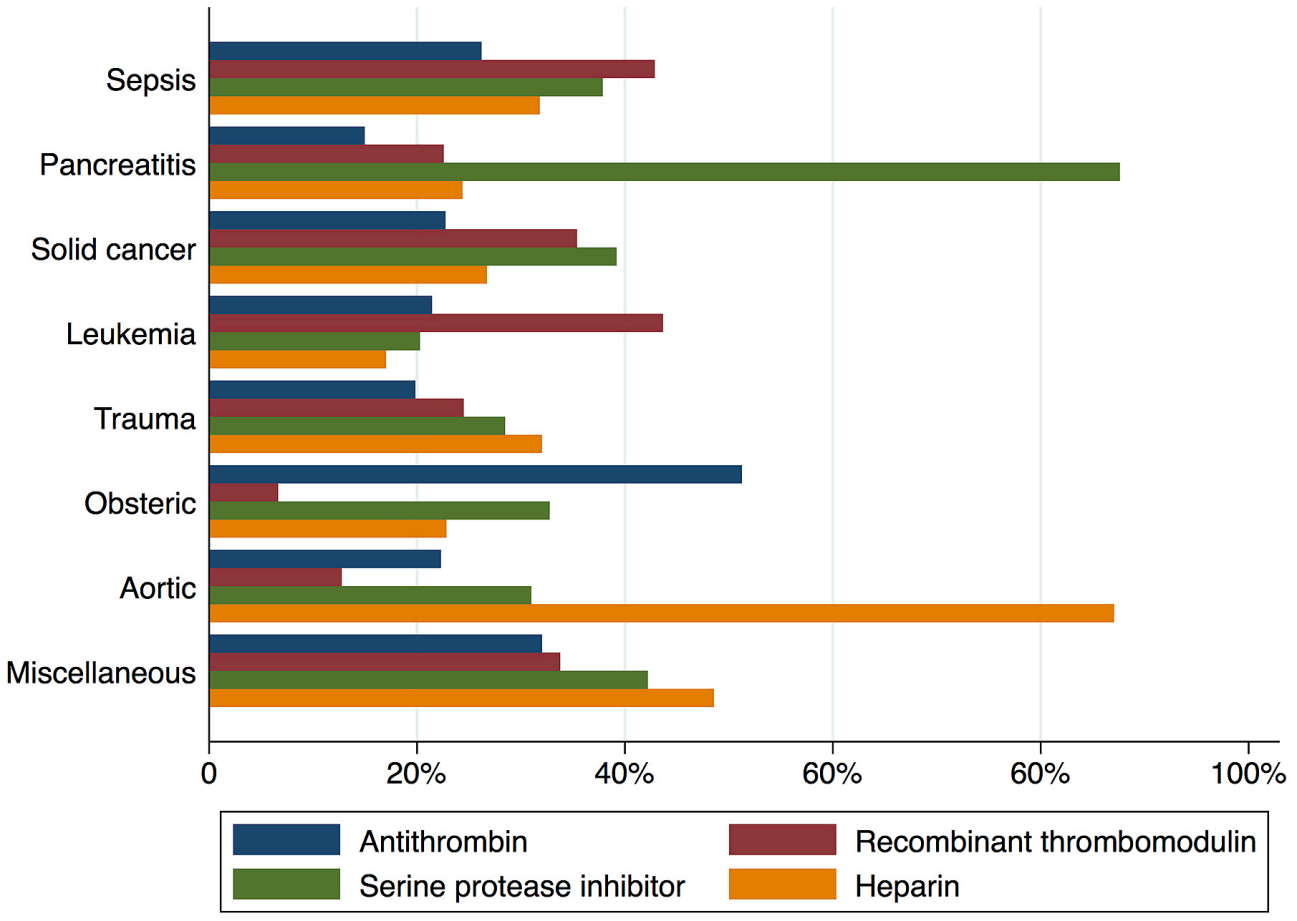
Use of anticoagulants for disseminated intravascular coagulation events by underlying disorders of disseminated intravascular coagulation.

**Figure 6.**
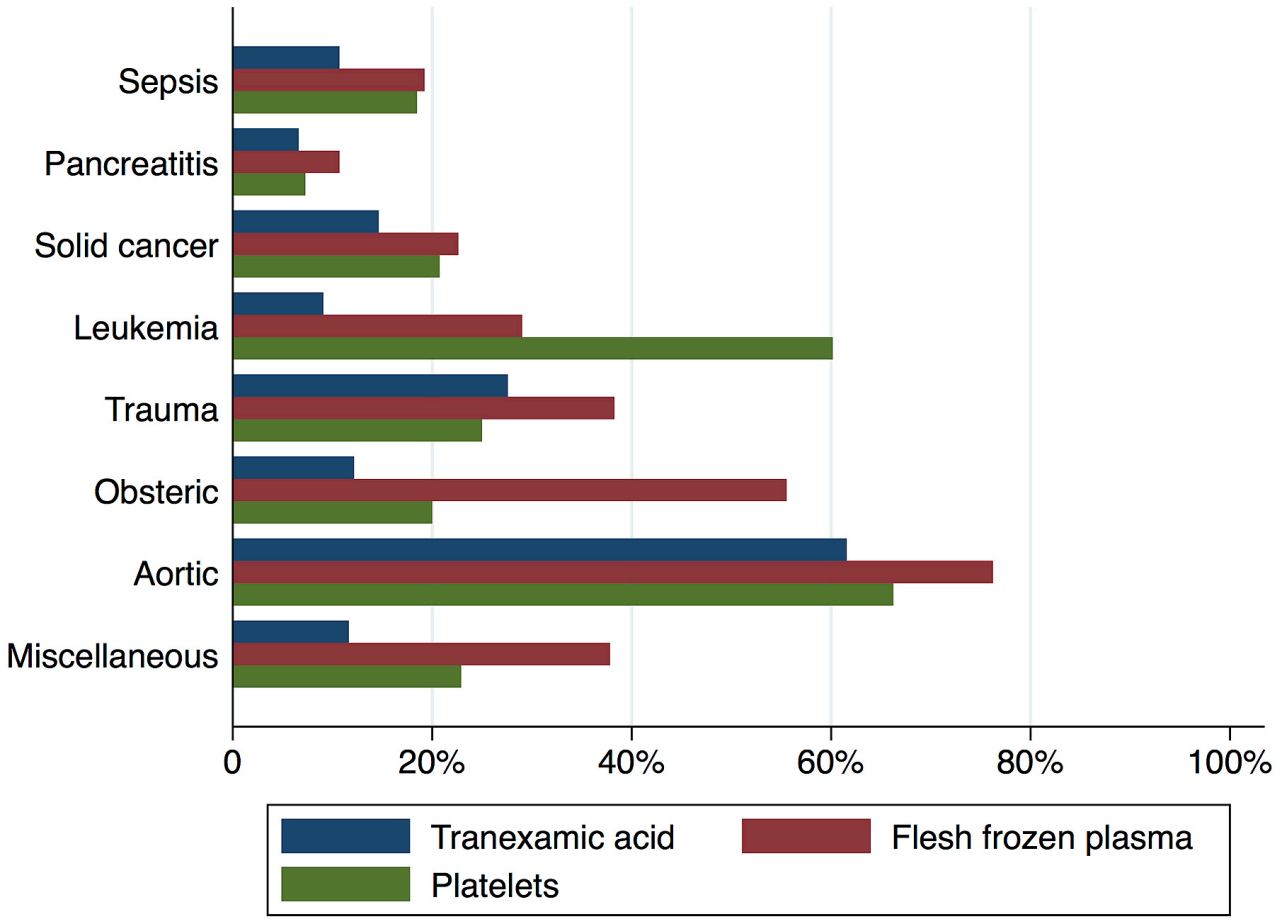
Use of antifibrinolytics and blood components for disseminated intravascular coagulation events by underlying disorders of disseminated intravascular coagulation.

Figure 2 shows in-hospital mortality by underlying disorders of DIC. In-hospital mortality exceeded 35% among patients whose underlying disorders were categorized as sepsis, solid cancer, leukemia, trauma, or miscellaneous. In contrast, in-hospital mortality was relatively low among patients with pancreatitis, obstetric diseases, and aortic diseases, at 16%, 0.6%, and 23.5%, respectively. Figure 3 presents the organ failure scores for patients with various underlying disorders of DIC. Higher organ failure scores were seen among patients with aortic diseases, sepsis, and trauma (means of 2.8, 2.2, and 2.2, respectively). Figure 4 shows major bleeding events by underlying disorders of DIC. Higher rates of major bleeding events were observed among patients with aortic diseases, trauma, obstetric diseases, and solid cancer (means of 24%, 15%, 10%, and 10%, respectively). Figure 5 shows the anticoagulants used for DIC treatment by underlying disorders of DIC. Antithrombin was used more frequently among patients with obstetric diseases or sepsis (51% and 26%, respectively). Recombinant thrombomodulin was used more often among patients with leukemia or sepsis (44% and 43%, respectively). Figure 6 shows the antifibrinolytics and blood components used for DIC treatment by underlying disorders of DIC. Tranexamic acid was used more often among patients with aortic diseases or trauma (62% and 28%, respectively). Fresh frozen plasma was more frequently used among patients with aortic diseases, obstetric diseases, trauma, or leukemia (76%, 56%, 38%, and 29%, respectively). Platelets were used more often among patients with aortic diseases and leukemia (66% and 60%, respectively).

## Discussion

Using a national inpatient database in Japan, our study analyzed a large number of patients with DIC and revealed the associations between underlying disorders, clinical phenotypes, and actual treatment patterns.

Our results suggest that the existing clinical phenotypes of DIC are not associated with the clinical presentation of organ failure or marked bleeding in patients with various underlying disorders. Resulting in organ failure, pathophysiologically all underlying disorders of DIC share the ability to induce systemic activation of coagulation ^(1)^. The clinical presentation of organ failure or marked bleeding is not caused only by DIC but also by each underlying disorder itself. The clinical presentation of DIC can shift or change over time, especially in patients with the complication of sepsis during hospitalization ^(11)^. Furthermore, because of the heterogeneity in fibrinolytic activity between cases, it is assumed that there are various phenotypes within the same underlying disorder.

Current clinical guidelines state that DIC treatment should be based on the clinical phenotypes ^(2), (6), (7), (8)^. However, it is difficult for clinicians to identify these phenotypes because the underlying disorders of DIC are not associated with the clinical presentation or existing phenotypes, and there is currently no gold standard for identifying phenotypes. Considering our results and the complicated mechanism of DIC, future research is necessary to develop new diagnostic tools to identify the phenotypes of DIC and to improve individual treatment strategies, for example, new diagnostic criteria for DIC from the Japanese Society on Thrombosis and Hemostasis suggest the use of several markers that may be able to classify the phenotypes of DIC ^(26)^.

Regardless of the underlying disorders, anticoagulants are widely used in the treatment of DIC, but the choice of anticoagulants varies across underlying disorders. For example, antithrombin use was higher in patients with obstetric diseases. This is because, on the basis of expert opinion, the Japanese guidelines for obstetric critical bleeding recommend the use of antithrombin for obstetric DIC. Another example is the higher use of recombinant thrombomodulin in patients with leukemia, which is seen because the Japanese guidelines recommend its use, again on the basis of expert opinion. Further studies are necessary among patients with various underlying disorders as, eexcept in the case of sepsis ^(27), (28)^, the current recommendations of anticoagulants for DIC are based on expert opinion or experimental use.

The present study had several limitations. Firstly, compared with the diagnoses in planned prospective studies, the diagnoses recorded in administrative claims databases are generally less accurate. However, in the database used in the present study, many inpatient diagnoses are known to have high specificity and positive predictive value ^(12), (13)^. Secondly, misclassification of underlying disorders may have occurred because we used a complicated classification process with a hierarchical system of diagnoses and procedures to create mutually exclusive groups. However, in this study, the incidence rates of disorders underlying DIC were similar to those reported in previous studies ^(11), (29)^. Furthermore, our findings for mortality from DIC were also similar to those in previous studies ^(11), (30)^. Thirdly, the diagnoses of DIC used in the present study were based on ICD-10 codes recorded by attending physicians, and it is possible that these diagnoses were not always based strictly on the diagnostic criteria from the Japanese Association for Acute Medicine or the International Society of Thrombosis and Haemostasis ^(2), (14)^. Fourthly, we did not have access to the results of coagulation tests, hemostatic molecular markers, or cytokines. Fifthly, we could not distinguish DIC-related organ failure from underlying disorders related organ failure or comorbidity related organ failure. Similarly, we could not distinguish DIC-related bleeding from thrombocytopenia-related bleeding, anti-coagulant therapy caused bleeding, or trauma-related bleeding. However, even when an accurate prospective study for DIC patients is performed, in general it is not possible to clearly distinguish the direct cause of organ failure and bleeding.

In conclusion, the present study suggests that the clinical presentations of bleeding and organ failure are not associated with the three existing clinical phenotypes of DIC or with the underlying disorders of DIC. Therefore, clinical presentation alone may not be sufficient for identifying the clinical phenotypes of DIC. Further research is necessary to develop new strategies for identifying the phenotypes of DIC and improving treatment strategies for individual patients.

## Article Information

### Conflicts of Interest

None

### Acknowledgement

The authors declare that they have no conflicts of interest. This work was supported by grants from the Ministry of Health, Labour and Welfare, Japan (19AA2007 and H30-Policy-Designated-004) and the Ministry of Education, Culture, Sports, Science and Technology, Japan (17H04141).

### Sources of Funding

This work was supported by the Ministry of Health, Labor and Welfare, Japan [grant numbers 19AA2007 and H30-Policy-Designated-004] and the Ministry of Education, Culture, Sports, Science and Technology, Japan [grant number 17H04141].

### Author Contributions

HO, KY, and KT designed the research; KM, HM and KF conducted the research; HO, KY, and KT analyzed the data; HO, KY, KT, and HY wrote the paper; and KY had primary responsibility for the final content. All authors read and approved the final manuscript.

### Approval by Institutional Review Board (IRB)

The study was approved by the Institutional Review Board of The University of Tokyo (approval number: 3501-[1] [July 25, 2011]).

## Supplement

Supplementary Table
