# Supplementary material for: Underlying Disorders, Clinical Phenotypes, and Treatment Diversity among Patients with Disseminated Intravascular Coagulation
Source: JMA J. 2020 Sep 23;3(4):321–9. doi: 10.31662/jmaj.2020-0023 (PMC7677446; doi:10.31662/jmaj.2020-0023)
Supplement: Supplementary file 1 — Supplementary Table [file 2433-3298-3-4-0321-s001.pdf]

Supplementary Table S1. Diagnosis and procedure code-based criteria used to define the underlying disorders of disseminated intravascular coagulation

Category	Code types	Codes	Related criteria
**Sepsis**	ICD-10 codes in the primary, concomitant, or complication diagnoses	A02, A04, A05, A08, A09, A15–A19, A27, A28, A31, A32, A35, A37, A39, A40–A43, A46, A48, A49, A52–A54, A69, B35, B36, B374–B379, B44–B46, B48, B49, G00–G09, I30, I33, I80, J01–J06, J13–J18, J20–J22, J440, J441, J47, J69, J85, J86, K35–K37, K57, K61, K630, K631, K65, K67, K750, K751, K80, K81, K830, K918, L00, L03, L04, L08, L88, M00, M86, N10–N12, N151, N159, N160, N30, N34, N390, N41, N70–N73, N76, N77, T814, T826, T827, T835, T845–T847, T857,	Absence of codes to qualify for any of the other seven categories
**Solid cancer**	ICD-10 codes in the primary, concomitant, or complication diagnoses	C00–C80, C97, D37–D48	Absence of codes for obstetric diseases, aortic diseases, or leukemia
**Leukemia**	ICD-10 codes in the primary, concomitant, or complication diagnoses	C81–C96	Absence of codes for obstetric diseases or aortic diseases
**Trauma**	ICD-10 codes in the primary, concomitant, or complication diagnoses	S00–T14	Absence of codes for obstetric diseases, aortic diseases, leukemia, solid cancer, or miscellaneous
**Obstetric diseases**	ICD-10 codes in the primary diagnoses	O00–O99	
	Japanese DPC code Form 1	Existence of pregnancy during hospitalization	
**Pancreatitis**	ICD-10 codes in the primary diagnoses	K85	Absence of codes for obstetric diseases, aortic diseases, leukemia, solid cancer, miscellaneous, or trauma
	Japanese DPC code Form 1	Existence of the acute pancreatitis severity score	
**Aortic diseases**	ICD-10 codes in the primary	I71	Absence of codes for obstetric

	diagnoses		diseases
	Japanese medical procedure codes	K560, K561	
**Miscellaneous**	ICD-10 codes in the primary diagnoses	D180, G210, I46, I490, I70, I72, I74, K550, M30, M310, M312–M319, M6289, N280, T20–T32, T630, T67, T68	Absence of codes for obstetric diseases, aortic diseases, leukemia, or solid cancer

ICD-10: International Classification of Diseases, Tenth Revision; DPC: Diagnosis Procedure Combination

Supplemental Table S2. ICD-10 codes for type of infection

Type of infection	ICD-10 codes
**Lung**	A15 A16 A19 A310 A37 B371 B440 B441 B450 J01–J06 J13–J18 J20–J22 J440 J441 J47 J85 J86
**Abdomen**	A020 A04 A05 A08 A09 K35–K37 K57 K61 K630 K631 K65 K67 K750 K751 K800 K803 K804 K808 K810 K830 K918
**Urinary tract**	A181 B374 N10–N12 N151 N159 N160 N30 N34 N390 N41 T835
**Central nervous system**	A17 A39 B375 G00–G09
**Skin and soft tissues**	A311 A35 A46 A480 A483 B35 B36 B372 L00 L03 L04 L08 L726 L88
**Cardiovascular system**	B376 I30 I33 I80 T826 T827
**Other infections**	ICD-10 codes for sepsis other than those for above six types of infection

ICD-10, International Classification of Diseases 10th revision

Supplemental Table S3. ICD-10 codes for type of solid cancer

Type of solid cancer	ICD-10 codes
**Esophagus**	C15
**Stomach**	C16
**Colon**	C18–C20
**Liver**	C22
**Bile duct/gallbladder**	C23 C24
**Pancreas**	C25
**Lung**	C33 C34 C37–C39
**Gynecological**	C53 C54 C56
**Urological**	C61 C64–C67
**Breast**	C50
**Other solid cancers**	ICD-10 codes for solid cancer other than those for above ten types of solid cancer

ICD-10, International Classification of Diseases 10th revision

Supplementary Table S4. ICD-10 codes and Japanese procedure codes used to calculate the organ failure score

Type of organ failure	ICD-10 codes	Japanese procedure codes
**Cardiovascular**	I95, R57	Vasopressor or inotrope (dopamine, dobutamine, epinephrine, norepinephrine, vasopressin)
**Respiratory**		Mechanical ventilation
**Neurologic**	F05, G93.4	
**Hematologic***	D65, D695, D696, D698, D699, O450, O460, O723, O081	
**Hepatic**	K72.0, K76.3	
**Renal**	N17	Renal replacement therapy

ICD-10: International Classification of Diseases, Tenth Revision

*All patients diagnosed with disseminated intravascular coagulation (ICD-10 code: D65)
